# Automated Online Quantification Method for ^18^F-FDG Positron Emission Tomography/CT Improves Detection of the Epileptogenic Zone in Patients with Pharmacoresistant Epilepsy

**DOI:** 10.3389/fneur.2017.00453

**Published:** 2017-09-01

**Authors:** Vanessa Cristina Mendes Coelho, Marcia E. Morita, Barbara J. Amorim, Celso Darío Ramos, Clarissa L. Yasuda, Helder Tedeschi, Enrico Ghizoni, Fernando Cendes

**Affiliations:** ^1^Neurology/Epilepsy, Unicamp – University of Campinas, Campinas, Brazil; ^2^Nuclear Medicine Department, Unicamp – University of Campinas, Campinas, Brazil; ^3^Neurosurgery/Epilepsy, Unicamp – University of Campinas, Campinas, Brazil

**Keywords:** epilepsy, focal cortical dysplasia, positron emission tomography imaging, quantification, automated data analysis

## Abstract

**Aims:**

To assess the validity of an online method to quantitatively evaluate cerebral hypometabolism in patients with pharmacoresistant focal epilepsy as a complement to the visual analysis of the ^18^F-FDG positron emission tomography (PET)/CT exam.

**Methods:**

A total of 39 patients with pharmacoresistant epilepsy and probable focal cortical dysplasia [22 patients with frontal lobe epilepsy (FLE) and 17 with temporal lobe epilepsy (TLE)] underwent a presurgical evaluation including EEG, video-EEG, MRI, and ^18^F-FDG PET/CT. We conducted the automated quantification of their ^18^F-FDG PET/CT data and compared the results with those of the visual-PET analysis conducted by experienced nuclear medicine physicians. For each patient group, we calculated Cohen’s Kappa coefficient for the visual and quantitative analyses, as well as each method’s sensitivity, specificity, and positive and negative predictive values.

**Results:**

For the TLE group, both the visual and quantitative analyses showed high agreement. Thus, although the quantitative analysis could be used as a complement, the visual analysis on its own was consistent and precise. For the FLE group, on the other hand, the visual analysis categorized almost half of the cases as normal, revealing very low agreement. For those patients, the quantitative analysis proved critical to identify the focal hypometabolism characteristic of the epileptogenic zone. Our results suggest that the quantitative analysis of ^18^F-FDG PET/CT data is critical for patients with extratemporal epilepsies, and especially those with subtle MRI findings. Furthermore, it can easily be used during the routine clinical evaluation of ^18^F-FDG PET/CT exams.

**Significance:**

Our results show that quantification of ^18^F-FDG PET is an informative complementary method that can be added to the routine visual evaluation of patients with subtle lesions, particularly those in the frontal lobes.

## Introduction

Epilepsy is a brain disorder characterized by persistent unprovoked seizures (1). Most patients diagnosed with epilepsy respond well to pharmacological treatment (2). How well a patient responds to treatment depends on the nature of the pathology as well as the complex interaction between genetic and environmental factors (3). Surgical treatment yields positive results in some forms of pharmacoresistant focal epilepsy, and success depends on the degree to which the epileptogenic zone (EZ) can be resected while preserving essential functional areas (4). In one recent study looking at focal cortical dysplasia (FCD), patients with Taylor-type dysplasia had more positive outcomes compared with patients with cytoarchitectural and architectural dysplasias (75 vs. 50 and 43% seizure-free, respectively) (5). Therefore, identifying the lesion responsible for seizure onset is critical for a better prognosis.

Lesion identification requires high-resolution imaging, image reconstructions, neurophysiological assessment, clinical and seizure semiology considerations, and an experienced neuroimager. Neuroimaging plays a particularly important role given that prognoses depend on accurate lesion visualization (6). However, the MRI images of 20–30% of temporal lobe epilepsy (TLE) patients and 20–40% of patients with extratemporal epilepsy do not show any abnormalities (7, 8).

Functional methods such as positron emission tomography (PET) are also used to detect epileptogenic lesions, and studies have found that the surgical prognosis of patients with well-defined lesions on MRI tends to be similar to that of patients whose PET scans reveal focal alterations (9, 10). ^18^F-FDG PET/CT localizes the EZ by detecting the hypometabolism of the dysfunctional (epileptogenic) neural tissue. However, since the area of hypometabolism usually extends beyond the EZ, ^18^F-FDG PET/CT usually lateralizes and localizes the epileptogenic area without clearly defining the borders of the dysplasia. Therefore, it is essential to correlate these data with other findings (11). Although the ideal analysis method for PET/CT has yet to be determined (12), the current gold standard is visual analysis, which has a sensitivity of 70–76% in TLE and 57–69% in extratemporal epilepsy (13–15). Visual analyses are highly observer-dependent, which may lead to false negatives. One way to reduce these is by utilizing complementary analysis methods.

Statistical Parametric Mapping (SPM; Wellcome Department of Clinical Neurology, Institute of Neurology, London, UK) is widely used to conduct automated and objective voxel-based data analyses in studies investigating various disorders, including epilepsy (16, 17). However, SPM analyses are somewhat complex and time-consuming, making them less than ideal as a complement to the visual analyses conducted during clinical routine. An alternative to this software is Scenium^®^ (from the Siemens Syngo.via Neurology software package); https://static.healthcare.siemens.com/siemens_hwem-hwem_ssxa_websites-context-root/wcm/idc/groups/public/@us/documents/download/mday/nziz/~edisp/scenium_creating_and_saving_key_images-01354774.pdf, which can be used to quantify brain hypometabolism during the time it takes to conduct the standard visual analysis. In this study, we choose Scenium^®^ software because it came with the purchased PET/CT equipment. While it was not originally used to perform quantitative analyses, during clinical assessments, we became aware of the fact that lateralization was identical in patients with well-defined lesions on MRI and on the visual analysis using PET/CT. This motivated us to test this software further. While there are other free software programs available, we did not conduct any systematic comparisons between them and Scenium.

Here, our goal was to determine how well this quantitative analysis could help identify subtle lesions on an individual basis.

## Materials and Methods

### Patient Characteristics

Patients were included in the study if they had refractory epilepsy and had potential or diagnosed FCD at the epilepsy outpatient clinic at Unicamp (Campinas, São Paulo, Brazil) between 2014 and 2016. We reviewed their medical records and collected the following data: epileptic seizure semiology, type of epilepsy, age at seizure onset, and frequency of seizures. We also collected EEG, video-EEG monitoring, brain MRI, and PET data. Patients with focal lesions and suspected FCD were classified according to the region of the probable EZ: (1) frontal lobe epilepsy (FLE) or (2) TLE. In a few cases where hypometabolism extended to the occipital or parietal lobes, patients were grouped according to the location of the main lesion/hypometabolic area (FLE or TLE; see Table 1 for details). Furthermore, in some cases, both the quantitative and qualitative analyses revealed bilateral alterations, precluding lateralization for those patients.

**Table 1:** Demographic and clinical patient data.

No.	Age	Sex	Seizure onset	EZ	Visual-positron emission tomography (PET)	EZ vs. visual-PET	Quantitative PET	Concordance: EZ vs. quantitative-PET	Surgical follow-up duration (months)	MRI	HP	Engel
1	14	M	3	R/FL	B/FL	Non-concordant	B/FL	Non-concordant		+	NO	
2	45	M	19	R/FL	NL	Non-concordant	R/FL	Concordant		−	NO	
3	15	F	13	R/FL	NL	Non-concordant	R/FL	Concordant	26	+	FCD II-B	II-A
4	24	F	22	R/FL	NL	Non-concordant	R/FL	Concordant	24	−/+	Gliosis	I-B
5	32	F	29	R/FL	NL	Non-concordant	B/FL	Non-concordant		−/+	NO	
6	13	F	4	R/FL	R/FL	Concordant	R/FL	Concordant	26	−/+	FCD II-A	III-A
7	32	F	26	R/FL	NL	Non-concordant	B/OL	Discordant	12	+	FCD II-A	IV-A
8	14	F	12	R/FL	R/FL	Concordant	R/FL	Concordant		−/+	NO	
9	23	M	5	R/FL	R/FL	Concordant	R/FL	Concordant		−/+	NO	
10	3	F	2	L/FL	L/FL	Concordant	L/FL	Concordant	21	+	FCD II-B	IV-A
11	6	F	4	L/FL	L/FL	Concordant	L/FL	Concordant	22	+	Angiocentric glioma^a^	I-A
12	39	F	12	L/FL	B/PL	Discordant	L/FL	Concordant		−/+	NO	
13	28	F	18	L/FL	B/PL	Discordant	L/FL	Concordant	15	−/+	FCD II-B	IV-A
14	32	M	13	R/FPL	B/PL	Discordant	L/FL	Discordant		+	NO	
15	31	F	18	L/FL	L/TPL	Discordant	L/FTPL	Concordant		−/+	NO	
16	62	F	49	R/FL	NL	Non-concordant	R/FL	Concordant		−/+	NO	
17	35	M	14	L/FL	NL	Non-concordant	L/FL	Concordant		−/+	NO	
18	20	F	9	L/FL	NL	Non-concordant	B > L/FL	Concordant	32	+	FCD II-B	I-A
19	44	M	26	R/FPL	NL	Non-concordant	R/PL	Concordant	27	+	FCD II-B	I-B
20	23	F	9	L/FPL	NL	Non-concordant	L/FPL	Concordant		−/+	NO	
21	43	M	27	L/FL	NL	Non-concordant	R/FL	Discordant		+	NO	
22	38	M	26	L/FL	NL	Non-concordant	B/FL	Non-concordant		−	NO	
23	23	F	8	L/TOL	R/FPL	Discordant	R/FL	Discordant		+	NO	
24	57	M	24	L/TL	NL	Non-concordant	L/FPL	Discordant		−	NO	
25	53	M	40	R/TL	R/FTPOL	Concordant	R/TL	Concordant		+	FCD II-B	SUDEP
26	47	F	33	R/TL	R/TL	Concordant	R/TL	Concordant	19	−/+	Gliosis	IV-A
27	24	F	23	R/TL	R/TL	Concordant	R/TL	Concordant		+	NO	
28	35	M	33	R/TL	R/TL	Concordant	R/TL	Concordant		−/+	NO	
29	22	M	14	R/TL	NL	Non-concordant	R/TL	Concordant		+	NO	
30	39	F	21	L/TL	L/TL	Concordant	L/TL	Concordant		−/+	NO	
31	33	M	7	L/TL	L/TL	Concordant	L/TL	Concordant		−/+	NO	
32	44	F	21	L/TL	L/TL	Concordant	L/TL	Concordant		−/+	NO	
33	24	M	14	L/TL	L/TL	Concordant	L/TL	Concordant		−	NO	
34	29	F	9	L/TL	L/TL	Concordant	L/TL	Concordant		−/+	NO	
35	25	M	20	L/TL	L/TL	Concordant	L/TL	Concordant		−	NO	
36	42	F	40	L/TOL	L/TL	Concordant	L/TL	Concordant		−/+	NO	
37	58	M	23	L/TL	NL	Non-concordant	L/TL	Concordant	38	+	Gliosis	II-A
38	27	M	18	L/TL	NL	Non-concordant	L/TL	Concordant		–	NO	
39	33	F	20	B/TL	NL	Non-concordant	NL	Non-concordant		−	NO	

*Patients 1–22 were classified as frontal lobe epilepsy (FLE) (including 3 FPL) and 23–39 were classified as temporal lobe epilepsy (including 2 TOL and 1 BTL). No., number; EZ, epileptogenic zone; visual-PET, visual PET data analysis; quantitative-PET, quantitative PET data analysis; MRI+, patients with lesions detected on MRI; MRI−, patient with negative MRI; MRI−/+, patient initially negative MRI and after functional images and/or new MRI sequences of became MRI+; NO, not operated; NL, normal; HP, histopathology; M, male; F, female; R, right; L, left; B, bilateral; FL, frontal lobe; BFL, bilateral frontal lobe; FPL, frontoparietal lobe; TL, temporal lobe; TOL, temporo-occipital lobe; PL, parietal lobe; OL, occipital lobe; FTPOL, fronto–temporal–parietal lobe; FCD, focal cortical dysplasia; SUDEP, sudden unexpected death*.

**Figure 1:**
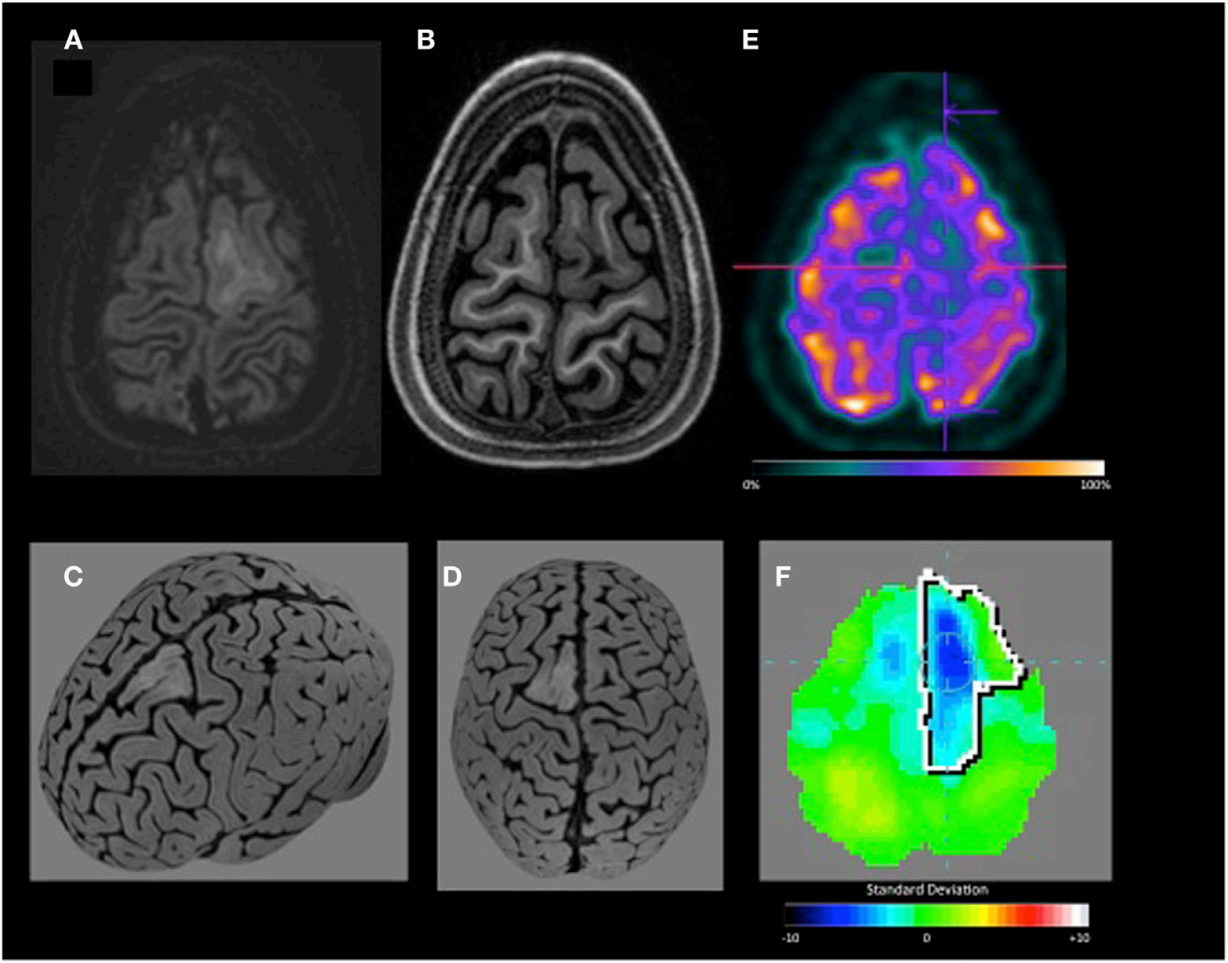
Left frontal lobe angiocentric glioma. This patient with histologically confirmed angiocentric glioma was included because focal cortical dysplasia was one of the differential diagnoses. **(A,B)** Double inversion recovery and T1 axial MRI show lesion in the left superior frontal gyrus, better characterized of **(C,D)** curvilinear reconstruction. **(E)** Axial visual-positron emission tomography (PET) shows hypometabolism in the left frontal. **(F)** Quantitative-PET confirms hypometabolism in the left frontal lobe with −4.3 SD.

*^a^This patient with histologically confirmed angiocentric glioma was included because FCD was one of the differential diagnoses as can be depicted in Figure 1*.

The EZ was defined according to the following criteria:

– In patients who underwent surgery for epilepsy, we considered the epileptogenic lesion to be the surgical gap (*n* = 12).
– In patients who did not undergo surgery:
  ◦ The EZ was defined as the FCD observed on the MRI that was concordant with the EEG and video-EEG (*n* = 6).
  ◦ In patients with negative MRI results and possible FCD, the EZ was defined based on seizure semiology, EEG, video-EEG, and a neuropsychological test (*n* = 21).

Following classification, we only included patients with probable or confirmed FCD with a frontal or temporal EZ who had undergone high-resolution MRI and PET at our center. Patients were excluded if they had dual pathology, preoperative diagnosis of tumor, sequelae of infarct or trauma, extensive cortical malformations, or an EZ entirely located outside the temporal or frontal lobes. We also excluded patients who underwent PET after the surgical procedure, since a surgical gap may alter the quantitative analysis of the hypometabolism. The final group consisted of 39 patients (mean age 29.5 years, range 3–62; 56.4% women). Of these, 17 were classified as TLE and 22 as FLE (see Table 1).

### Image and Data Analysis

The qualitative (visual) analysis was conducted by two experienced nuclear medicine doctors during the routine clinical evaluation of PET–CT scans. The raters had access to the clinical summary of the patients’ history and results of interictal EEGs, but not of the evaluation of high-resolution MRI. We classified the areas of cerebral hypometabolism detected by visual analysis as either normal or abnormal, in this case, we defined the location of the hypometabolism. When there was a disagreement, the final decision was made by consensus.

We conducted the quantitative analysis of the PET-scan images using Scenium^®^, a Siemens software program that is part of the Syngo.via Neurology package (Siemens CTI Molecular Imaging, Knoxville, TN, USA). Because this tool was initially developed to evaluate patients with dementia, the control group consists of individuals 46–79 years old. Once control individuals have been chosen from the databank, the software fuses each patient’s MRI with that of the software by placing both in the same standardized space. Finally, the software conducts a voxel-by-voxel statistical comparison of each patient’s PET scan to that of the control group. This is an automated quantification and thus does not depend on the operator. The quantitative analysis delineates areas of significant hypometabolism (>2 SDs from the mean) and depicts them in images as well as in a table. The regions of hypo- and hypermetabolism are displayed in different colors, and the region with the greatest hypometabolism is defined.

We then compared the EZ with the PET–CT hypometabolism. In comparison to the EZ, we defined the PET visual and quantitative analyses as: (i) *concordant*, when there was an overlap between the hypometabolism and EZ, or when there was bilateral asymmetric hypometabolism with predominance over the EZ. In the case of quantitative PET analysis, we considered as concordant the area of significant hypometabolism (>2 SDs) delineated by the software or, when bilateral asymmetric, the area with most extensive and intense hypometabolism defined as the maximum of SD within the area of abnormality (see for example Figure 1); (ii) *Non-concordant*, when the PET analyses were negative or there was a bilateral symmetrical hypometabolism; and (iii) *Discordant*, when there was a hypometabolism contralateral or localized in a different lobe and not involving the EZ. For statistical analyses, we lumped together the non-concordant and discordant groups. When patients had a negative MRI, the analysis of concordance was assessed based on clinical data (seizure semiology, EEG, video-EEG, and a neuropsychological test).

Since hypometabolism usually extends to the area surrounding the EZ, we considered the EZ concordant with the visual PET analysis (visual-PET) or quantitative-PET analysis (quantitative-PET) if the maximal hypometabolic area overlapped with the EZ, even if it extended to multiple lobes. In case of bilateral hypometabolism in homologous areas, if the EZ overlapped the more extensive hypometabolism area, we interpreted this finding as propagation and considered this case concordant.

In each group (TLE and FLE), we conducted two concordance analyses using the Kappa index. These were:

– EZ × the area suggested by the visual-PET
– EZ × the area detected by the quantitative-PET

Results were considered “positive” when the established EZ was concordant with the visual or quantitative analysis results.

According to Landis and Koch (18), Kappa values are classified as: slight (0.00–0.20), fair (0.21–0.40), moderate (0.41–0.60), substantial (0.61–0.80), and almost perfect (0.81–1.00).

In addition, for each patient group (TLE and FLE), we calculated each analysis method’s (visual vs. quantitative) sensitivity, specificity, positive predictive value (PPV), and negative predictive value (Table 2).

**Table 2:** Summary of visual and quantitative positron emission tomography analyses as compared to the location of the epileptogenic zone.

	Concordant	*K*	Sensitivity	Specificity	PPV	NPV	Non-concordant or discordant
TLE visual	11	0.62	0.64	0.95	0.91	0.77	6
TLE quantitative	14	0.78	0.82	0.95	0.93	0.87	3
FLE visual	5	0.11	0.22	0.88	0.71	0.46	17
FLE quantitative	16	0.59	0.72	0.88	0.88	0.71	6

*PPV, positive predictive value; NPV, negative predictive value; TLE, temporal lobe epilepsy; FLE, frontal lobe epilepsy*.

## Results

(1) Patients with TLE

When we compared the EZ in the temporal lobe to the area suggested by the visual analysis of the FDG-PET data, we observed that 11/17 patients (64.7%) were concordant, with a Kappa index value of 0.62 [considered substantial, according to Landis and Koch (18)].

When we then compared the EZ in the temporal lobe to the area suggested by the quantitative-PET, we observed that 14/17 patients (82.3%) were concordant, with a Kappa index value of 0.78 (also considered substantial).

(2) Patients with FLE

When we compared the EZ in the frontal lobe to the area suggested by the visual-PET, we observed that 5/22 patients (22.7%) were concordant, with a Kappa index value of 0.11 (considered slight).

When we then compared the EZ in the frontal lobe to the area suggested by the quantitative-PET, we observed that 16/22 patients (72.7%) were concordant, with a Kappa index value of 0.59 (considered moderate).

Of the 12 patients who had surgery, four patients had negative MRI on the first evaluation, but after visual and quantitative PET analyses, MRIs were re-evaluated, including new sequences [e.g., double inversion recovery (DIR)], and the MRIs became positive (see Table 1). From these, only one had Engel I outcome. Patient 10 (classified as DCF II-B) had an extensive lesion affecting functional eloquent areas (motor and language), which prevented complete lesion resection. Although classified as DCF II-B, patient 13 initially presented subtle imaging findings, and most likely continued experiencing seizures due to incomplete lesion resection.

Six patients with lesions detected on MRI did not have surgery. One patient is waiting for surgery to be performed, two decided to postpone surgery and three are still under investigation.

The inclusion of the software analysis added only a few minutes to the PET reviewing time. It was user friendly and well accepted by the nuclear medicine team.

## Discussion

**Figure 2:**
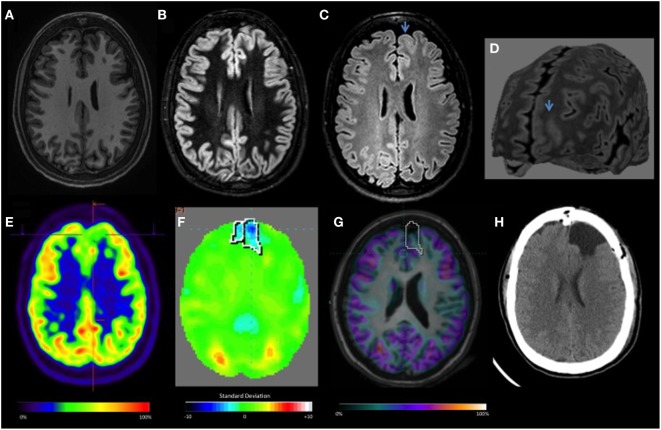
Focal cortical dysplasia II-B left frontal lobe: **(A–D)** T1, double inversion recovery, FLAIR axial MRI, and curvilinear reconstruction, showing abnormal cortical thickening, focal increased signal, white and gray matter junction blurred (arrow). **(E)** Visual-positron emission tomography (PET) initially was considered normal. **(F)** Quantitative-PET showed −3.6 SD in the left frontal lobe. **(G)** Co-registration MRI and PET. **(H)** Axial CT after surgery resection.

Our goals in this study were to evaluate whether (1) online quantification is a useful complement to the visual analysis of ^18^F-FDG PET/CT exams and (2) whether the software we used (Scenium^®^) yields robust results. Our results suggest that the quantitative-PET is critical for patients with extratemporal epilepsies and subtle MR findings (Figure 2). After trying the ^18^F-FDG PET/CT quantification tool, we considered that it could be easily implemented during routine clinical analysis of patients with focal epilepsy, since it did not add a lot of processing time and effort.

**Figure 3:**
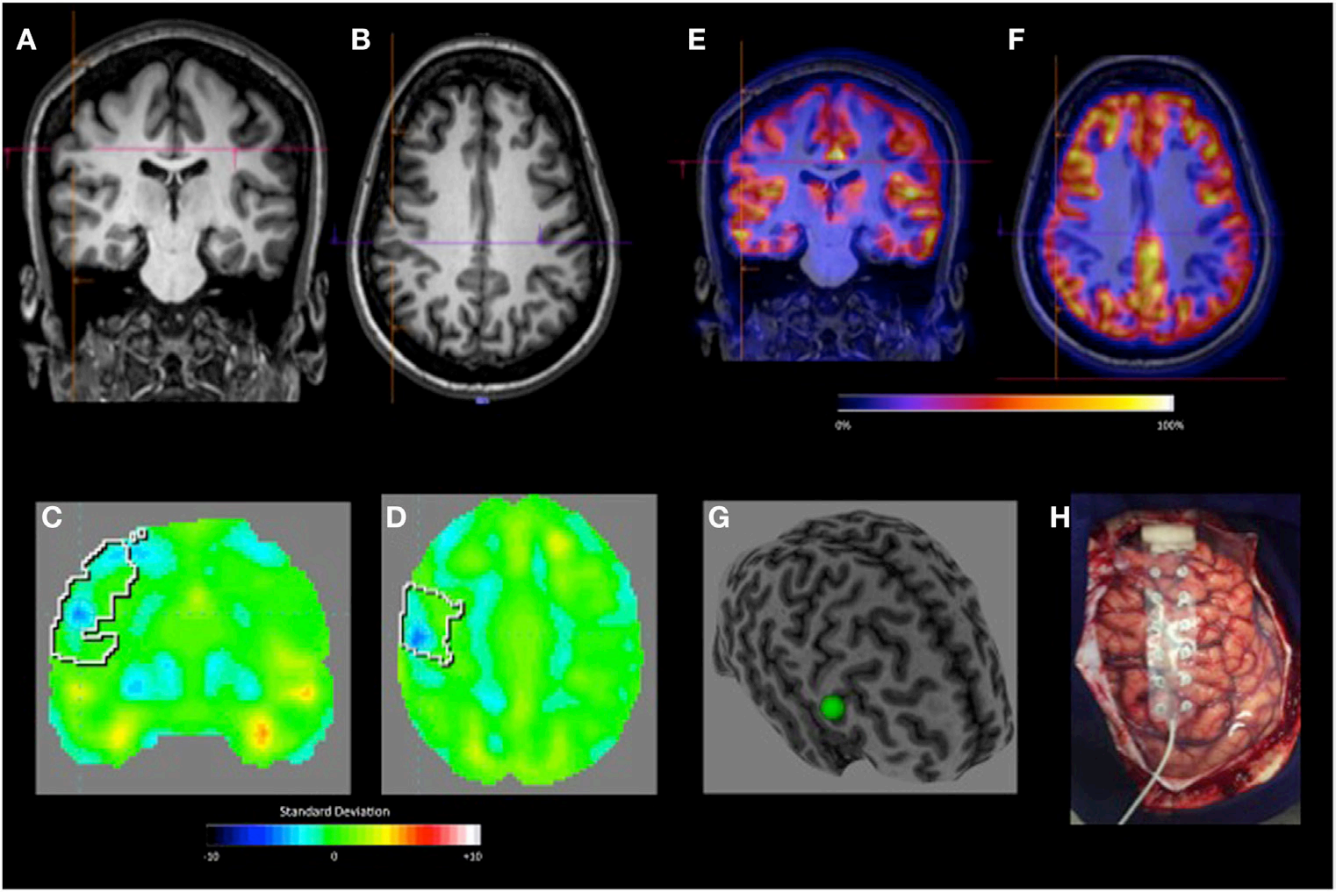
Gliosis (oligodendrogial hyperplasia). **(A,B)** Coronal and axial negative-MRI. The visual-positron emission tomography (PET) was initially considered normal. **(C,D)** Quantitative-PET showing hypometabolic area in the right central area with −4.8 SD. **(E,F)** Coronal and axial PET/MRI co-registration. **(G)** Curvilinear reconstruction show discrete convergence of sulci and gyrus. **(H)** Resected brain tissue.

The main method currently used to evaluate ^18^F-FDG PET/CT studies is visual interpretation, which is operator-dependent (19). Thus, subtle or focal alterations may go unnoticed or oftentimes classified as normal (Figure 3). In this study, despite the nuclear medicine physician’s experience, almost half of the studied patients with refractory epilepsy (13 FLE and 4 TLE) were classified as normal by the visual analysis. After quantification of the ^18^F-FDG PET/CT data, 52% of those patients with previously normal PET scans became “positive,” and hypometabolism was confirmed in the predetermined EZ. The quantitative-PET was particularly useful in the detection of frontal lobe lesions, which were relatively more subtle and difficult to detect *via* visual analysis. Therefore, the present study shows that complementing the traditional method of visual interpretation of FDG-PET with automatic quantification contributes to the detection of hypometabolic areas in patients with possible refractory FCD, especially those with lesions located in the frontal lobe. In fact, a second PET visual analysis was conducted after quantitative analysis and could identify the hypometabolic areas that were missed during the first in 11 (50%) of patients with FLE and 3 (18.7%) of patients with TLE.

For the TLE group, both PET analyses detected abnormalities in a similar proportion of patients. This suggests that, although quantification may also offer complementary information for this group, the visual analysis may be enough on its own. Unlike the temporal lobe, where a more restricted area allows for better-defined alterations, the frontal lobe harbors epileptogenic foci that may propagate over a larger region, thus resulting in more tenuous focal cerebral hypometabolism.

Patients in this study were characterized by their resistance to clinical treatment and possible FCD. Most patients (59% of FLE and 70% of TLE) did not show a significant lesion on MRI and some of them (12/22; mostly those with a probable frontal lesion) also did not show any hypometabolism on the visual analysis. In those cases, the quantitative-PET was critical for the localization and lateralization of their lesions. These findings are in line with the previously described notion that frontal alterations may be too subtle to detect on both MRI and PET (20–23).

Focal cortical dysplasia is the second most common cause of pharmacoresistant epilepsy and sometimes surgery offers the only possibility of remission. Adequately resecting the dysfunctional neural tissue responsible for seizures is critical for a good prognosis (4). Therefore, it is essential that we detect and characterize these lesions prior to surgery. MRI is the “gold standard” method to detect these lesions, yet, 20–30% of patients with temporal epilepsy and 20–40% of those with extratemporal epilepsy do not show well-defined alterations on MRI. ^18^F-FDG PET/CT, on the other hand, can provide information regarding localization or lateralization of the epileptogenic focus in 60–90% of patients with temporal epilepsy and 30–60% of patients with extratemporal epilepsy (24), with surgical outcomes similar to those with positive MRI. These numbers highlight the need to develop methods that can fill the gap left by visual analyses of MRI and ^18^F-FDG PET/CT scans.

For patients with normal MRI who underwent surgery, the combined PET, EEG, and clinical semiology results led to the decision to perform surgery. The surgical failure is most likely related to the extent of the lesion, which was not adequately defined by either MRI or PET or due to the involvement of eloquent areas that could not be resected.

In the current study, automated quantification with Scenium^®^ reliably detected PET hypometabolic areas, which were verified by the presence of well-defined lesions on MRI (Figure 1) and/or by the histology results of the resected brain tissue. While software programs such as SPM (Wellcome Department of Clinical Neurology, Institute of Neurology, London, UK) are widely used and also yield satisfactory quantification results (17, 25), analyses are often complex and time consuming, making them impractical as an online complement to the visual analyses conducted during routine clinical practice. In a recent study, Wang et al. (26) compared epileptic focus detection by SPM and 3D-SSP for 18F-FDG-PET brain mapping analyses and concluded that both methods can improve the detection rate and recommended the use of 3D-SPP, and SPM for more complex cases. However, they included mainly cases with TLE with hippocampal sclerosis. In our study, we emphasize the usefulness of quantification in difficult cases such as negative MRI or subtle FCD, excluding cases of hippocampal sclerosis. In summary, our study agrees with the literature that regardless the method (different technics), quantification of PET scan is a useful tool.

**Figure 4:**
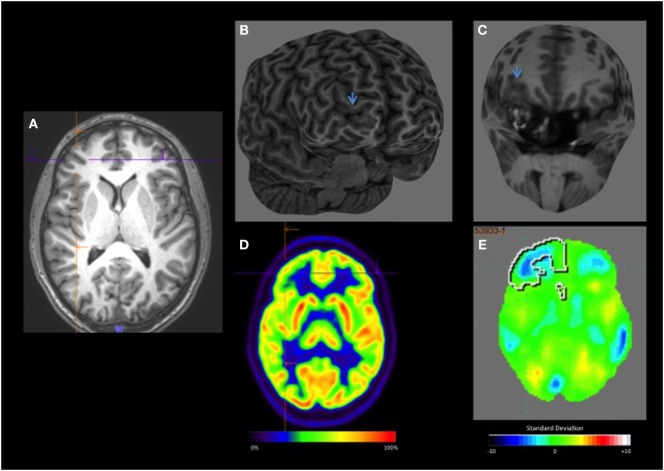
**(A)** Axial T1 negative-MRI **(B,C)** curvilinear reconstruction shows better the cortical thickening and abnormalities of sulcus and gyrus pattern in the right anterior frontal lobe. **(D)** visual-positron emission tomography (PET) showing slight hypometabolism in the right frontal lobe. **(E)** Quantitative-PET shows hypometabolism in the right frontal lobe −3.7 SD.

Thus, one critical advantage of conducting quantification using Scenium^®^ is that it can be done during clinical practice, offering dynamic and individualized evaluations (Figure 4). Furthermore, unlike analyses using software programs such as SPM, this type of analysis fits in well with the nuclear medicine physician’s normal routine.

The current study had some limitations. First, the small number of patients who underwent surgery could be considered a major limitation in determining the reliability of this method in accurately defining the EZ; however, we have to keep in mind that these are very difficult cases and any additional information or non-invasive tool could be of great importance in this setting. Especially for patients not submitted to surgery and with negative MRI, the analysis of concordance is an even more difficult task, but by giving additional information on lateralization and localization, especially when the area of hypometabolism is concordant with the suspected area, it reassures us that we are following the right track.

Another limitation is that the Scenium^®^ control group is somewhat older than the individuals tested in our study. This means that milder abnormalities in the experimental group may have gone unnoticed, as they would not appear to be significantly different from the relatively more widespread hypometabolism typical of older individuals. This should be taken into account when interpreting the results. Furthermore, some patients with positive MRI results had inconclusive quantification results; this most likely reflects bilateral hypometabolism with similar SD values, which reflects the network dysfunction typical of focal epilepsies. Finally, the quantitative analysis described here requires the Scenium^®^ software, which may not be available to every hospital or clinical group (27).

In conclusion, the quantitative analysis of glucose metabolism in ^18^F-FDG PET/CT images is useful in cases that are inconclusive and can help define the EZ when MRI fails. Furthermore, implementing automated quantitative techniques to complement the visual analysis of PET images can significantly improve the method’s sensitivity and utility. Scenium^®^ is a relatively easy to use and highly useful tool, as long as the clinician or investigator conducts a thorough and careful analysis that also considers clinical and EEG data, as well as the extent of hypometabolism. Future studies with larger cohorts should be conducted to further test the present findings. Additional support for these data could help more centers gain access to the quantitative model and software to complement and improve their own clinical analyses.

## Ethics Statement

This study was approved by the institution’s Ethics Committee.

## Author Contributions

VC: conducted data collection, data analyses and wrote the manuscript. MM: contributed with study design and manuscript preparation and revision. BA and CR: contributed with PET data acquisition, analysis, and quantification. CY: contributed with study design, analyses of data, manuscript preparation and revision. HT and EG: neurosurgeons who conducted all surgeries and data collection. FC: contributed with study design and organization, funding, data analyses, manuscript preparation and revision.

## Conflict of Interest Statement

The authors declare that the research was conducted in the absence of any commercial or financial relationships that could be construed as a potential conflict of interest.

## References

[1] Duncan JS Sander JW Sisodiya SM Walker MC. Adult epilepsy. Lancet (2006) 367(9516):1087–100. 10.1016/S0140-6736(06)68477-8 16581409

[2] Kwan P Brodie MJ. Early identification of refractory epilepsy. N Engl J Med (2000) 342(5):314–9. 10.1056/NEJM200002033420503 10660394

[3] Cendes F Neuroimaging predictors of AED resistance in new-onset epilepsies. Epilepsia (2011) 52(S4):7–9. 10.1111/j.1528-1167.2011.03143.x 21732933

[4] Awad IA Rosenfeld J Ahl J Hahn JF Lüders H. Intractable epilepsy and structural lesions of the brain: mapping, resection strategies, and seizure outcome. Epilepsia (1991) 32(2):179–86. 10.1111/j.1528-1157.1991.tb05242.x 1900789

[5] Tassi L Colombo N Garbelli R Francione S Lo Russo G Mai R Focal cortical dysplasia: neuropathological subtypes, EEG, neuroimaging and surgical outcome. Brain (2002) 125(8):1719–32. 10.1093/brain/awf175 12135964

[6] Téllez-Zenteno JF Dhar R Wiebe S. Long-term seizure outcomes following epilepsy surgery: a systematic review and meta-analysis. Brain (2005) 128(5):1188–98. 10.1093/brain/awh449 15758038

[7] Carne RP O’Brien TJ Kilpatrick CJ MacGregor LR Hicks RJ Murphy MA MRI-negative PET-positive temporal lobe epilepsy: a distinct surgically remediable syndrome. Brain (2004) 127:2276–85. 10.1093/brain/awh257 15282217

[8] Hong KS Lee SK Kim JY Lee DS Chung CK. Pre-surgical evaluation and surgical outcome of 41 patients with non-lesional neocortical epilepsy. Seizure (2002) 11:184–92. 10.1053/seiz.2001.0616 12018962

[9] Gok B Jallo G Hayeri R Wahl R Aygun N. The evaluation of FDG-PET imaging for epileptogenic focus localization in patients with MRI positive and MRI negative temporal lobe epilepsy. Neuroradiology (2013) 55(5):541–50. 10.1007/s00234-012-1121-x 23223825

[10] LoPinto-Khoury C Sperling MR Skidmore C Nei M Evans J Sharan A Surgical outcome in PET-positive, MRI-negative patients with temporal lobe epilepsy. Epilepsia (2012) 53(2):342–8. 10.1111/j.1528-1167.2011.03359.x 22192050

[11] Hartl E Rémi J Vollmar C Goc J Loesch AM Rominger A PET imaging in extratemporal epilepsy requires consideration of electroclinical findings. Epilepsy Res (2016) 125:72–6. 10.1016/j.eplepsyres.2016.05.010 27399879

[12] Mazziotta JC Gilman S Clinical Brain Imaging: Principles and Applications (Vol. 39). USA: Oxford University Press (1992).

[13] Chandra PS Vaghania G Bal CS Tripathi M Kuruwale N Arora A Role of concordance between ictal-subtracted SPECT and PET in predicting long-term outcomes after epilepsy surgery. Epilepsy Res (2014) 108(10):1782–9. 10.1016/j.eplepsyres.2014.09.024 25308754

[14] van’t Klooster MA Huiskamp G Zijlmans M Debets RMC Comans EF Bouvard S Can we increase the yield of FDG-PET in the preoperative work-up for epilepsy surgery? Epilepsy Res (2014) 108(6):1095–105. 10.1016/j.eplepsyres.2014.04.011 24893829

[15] Brodbeck V Spinelli L Lascano AM Wissmeier M Vargas MI Vulliemoz S Electroencephalographic source imaging: a prospective study of 152 operated epileptic patients. Brain (2011) 134(10):2887–97. 10.1093/brain/awr243 21975586 PMC3187544

[16] Mayoral M Marti-Fuster B Carreño M Carrasco JL Bargalló N Donaire A Seizure-onset zone localization by statistical parametric mapping in visually normal (18) F-FDG PET studies. Epilepsia (2016) 57(8):1236–44. 10.1111/epi.13427 27286896

[17] Kim YK Lee DS Lee SK Chung CK Chung J Lee MC PET in localization of frontal lobe epilepsy: comparison of visual and SPM analysis. J Nucl Med (2002) 43(9):1167–74. 12215554

[18] Landis JR Koch GG. The measurement of observer agreement for categorical data. Biometrics (1977) 33:159–74. 10.2307/2529310 843571

[19] Drzezga A Arnold S Minoshima S Noachtar S Szecsi J Winkler P 18F-FDG PET studies in patients with extratemporal and temporal epilepsy: evaluation of an observer-independent analysis. J Nucl Med (1999) 40:737–46. 10319744

[20] Won HJ Chang KH Cheon JE Kim HD Lee DS Han MH Comparison of MR imaging with PET and Ictal SPECT in 118 patients with intractable epilepsy. AJNR Am J Neuroradiol (1999) 20(4):593–9. 10319968 PMC7056008

[21] Sadzot B Debets R Maquet P Comar C Franck G. PET studies of patients with partial epilepsy: visual interpretation vs. semi-quantification/quantification. Acta Neurol Scand Suppl (1994) 152:175–8. 10.1111/j.1600-0404.1994.tb05216.x 8209641

[22] Kim SK Na DG Byun HS Kim SE Suh YL Choi JY Focal cortical dysplasia: comparison of MRI and FDG-PET. J Comput Assist Tomogr (2000) 24(2):296–302. 10.1097/00004728-200003000-00022 10752897

[23] Lüders HO Awad IA Conceptual considerations. In: Lüders HO, editor. Epilepsy Surgery. New York: Raven Press (1991). p. 51–62.

[24] Chassoux F Rodrigo S Semah F Beuvon F Landre E Devaux B FDG-PET improves surgical outcome in negative MRI Taylor-type focal cortical dysplasias. Neurology (2010) 75:2168–75. 10.1212/WNL.0b013e31820203a9 21172840

[25] Van Bogaert P Massager N Tugendhaft P Wikler D Damhaut P Levivier M Statistical parametric mapping of regional glucose metabolism in mesial temporal lobe epilepsy. Neuroimage (2000) 12(2):129–38. 10.1006/nimg.2000.0606 10913319

[26] Wang K Liu T Zhao X Xia X Zhang K Qiao H Comparative study of voxel-based epileptic foci localization accuracy between statistical parametric mapping and three-dimensional stereotactic surface projection. Front Neurol (2016) 7:164. 10.3389/fneur.2016.00164 27729898 PMC5037321

[27] Bernhardt BC Bonilha L Gross DW. Network analysis for a network disorder: the emerging role of graph theory in the study of epilepsy. Epilepsy Behav (2015) 50:162–70. 10.1016/j.yebeh.2015.06.005 26159729

